# Root mediated uptake of *Salmonella* is different from phyto-pathogen and associated with the colonization of edible organs

**DOI:** 10.1186/s12870-018-1578-9

**Published:** 2018-12-11

**Authors:** Kapudeep Karmakar, Utpal Nath, Karaba N. Nataraja, Dipshikha Chakravortty

**Affiliations:** 1 Department of Microbiology and Cell Biology, Indian Institute of Science, Bangalore, 560012 India; 2 Centre for Biosystems Science and Engineering, Indian Institute of Science, Bangalore, 560012 India; 3 Department of Crop Physiology, University of Agricultural Science, GKVK, Bangalore, 560065 India

**Keywords:** Lateral roots, Epidermis remodeling, *Salmonella*, Tomato, Salinity

## Abstract

**Background:**

Pre-harvest contamination of fruits and vegetables by *Salmonella* in fields is one of the causes of food-borne outbreaks. Natural openings like stomata, hydathodes and fruit cracks are known to serve as entry points. While there are reports indicating that *Salmonella* colonize and enter root through lateral root emerging area, further investigations regarding how the accessibility of *Salmonella* to lateral root is different from phyto-pathogenic bacteria, the efficacy of lateral root to facilitate entry have remained unexplored. In this study we attempted to investigate the lateral root mediated entry of *Salmonella*, and to bridge this gap in knowledge.

**Results:**

Unlike phytopathogens, *Salmonella* cannot utilize cellulose as the sole carbon source. This negates the fact of active entry by degrading plant cellulose and pectin. Endophytic *Salmonella* colonization showed a high correlation with number of lateral roots. When given equal opportunity to colonize the plants with high or low lateral roots, *Salmonella* internalization was found higher in the plants with more lateral roots. However, the epiphytic colonization in both these plants remained unaltered. To understand the ecological significance, we induced lateral root production by increasing soil salinity which made the plants susceptible to *Salmonella* invasion and the plants showed higher *Salmonella* burden in the aerial organs.

**Conclusion:**

*Salmonella*, being unable to degrade plant cell wall material relies heavily on natural openings. Therefore, its invasion is highly dependent on the number of lateral roots which provides an entry point because of the epidermis remodeling. Thus, when number of lateral root was enhanced by increasing the soil salinity, plants became susceptible to *Salmonella* invasion in roots and its transmission to aerial organs.

**Electronic supplementary material:**

The online version of this article (10.1186/s12870-018-1578-9) contains supplementary material, which is available to authorized users.

## Background

*Salmonella* serovars are recognized as important food-borne pathogens associated with poultry [1, 2] and raw plant products [3, 4]. There has been an increase in human infections with various serovars linked to raw produce [5]. The raw plant products get contaminated during shipping and processing [6]. However, in the last few decades, reports have shown the evidence of pre-harvest contamination of salad vegetables [7, 8]. Irrespective of the species, plants grown in *Salmonella*-contaminated soil became colonized with the organism [9, 10].

Various biotic and abiotic components play an important role in successful colonization of *Salmonella* in roots. *De-novo* factors like flagella [11], fimbriae [12], and exopolysaccharides [13] are known to enable pathogen to colonize the host. Presence of a phytopathogen like *Xanthomonas* [14] in vicinity can lead to higher colonization of *Salmonella*. But, beneficial organisms like *Sinorhizobium meliloti* is known to reduce the burden of *Salmonella* in plants [15]. Gu et al reported the presence of *Salmonella* in the vasculature of the leaves and the fruits without causing any visible symptoms [16].

The entry point of *Salmonella* in plants is well-studied. They can enter the aerial organs through the openings such as stomata [17], hydathodes [18] and fruit cracks [19]. Unlike aerial organs, below ground organs are in direct contact with the contaminated soil. *Salmonella,* can become systemic after its entry into the plant and can colonize the aerial organs [20, 21]. Cooley et al. has shown the movement of *Salmonella* Newport and *E.coli* O157:H7 as an epiphytic migrant. They have also reported that *E.coli* O157:H7 can enter the vasculature of the plant but not *Salmonella* Newport. Lateral root emerging areas with epidermal breakage, were shown to be colonized with these organisms and flagella aids in migration to these regions [22]. Many bacteria and fungi utilize this opening for getting access to root tissues [23, 24]. While there are reports indicating that lateral root emerging regions are the site of *Salmonella* entry and colonization [22, 25], the mechanism of entry is not well explored. Investigations regarding how the accessibility to lateral root for *Salmonella* is different from phyto-pathogens and the possibilities such as specific induction of lateral root formation by the bacteria have remained unexplored. The aim of the study is to understand the role of the lateral root in mediating *Salmonella* entry in a more elusive way. We used wild-type Arabidopsis with Col-0 accession and tomato (cultivar INDAM 535) as plant model systems. As an extension of the study, we have examined soil stress factor (salinity) in transmission of *Salmonella* from soil to the aerial organs.

## Results

### *Salmonella* colonization of root is different from phyto-pathogen colonization

**Fig. 1:**
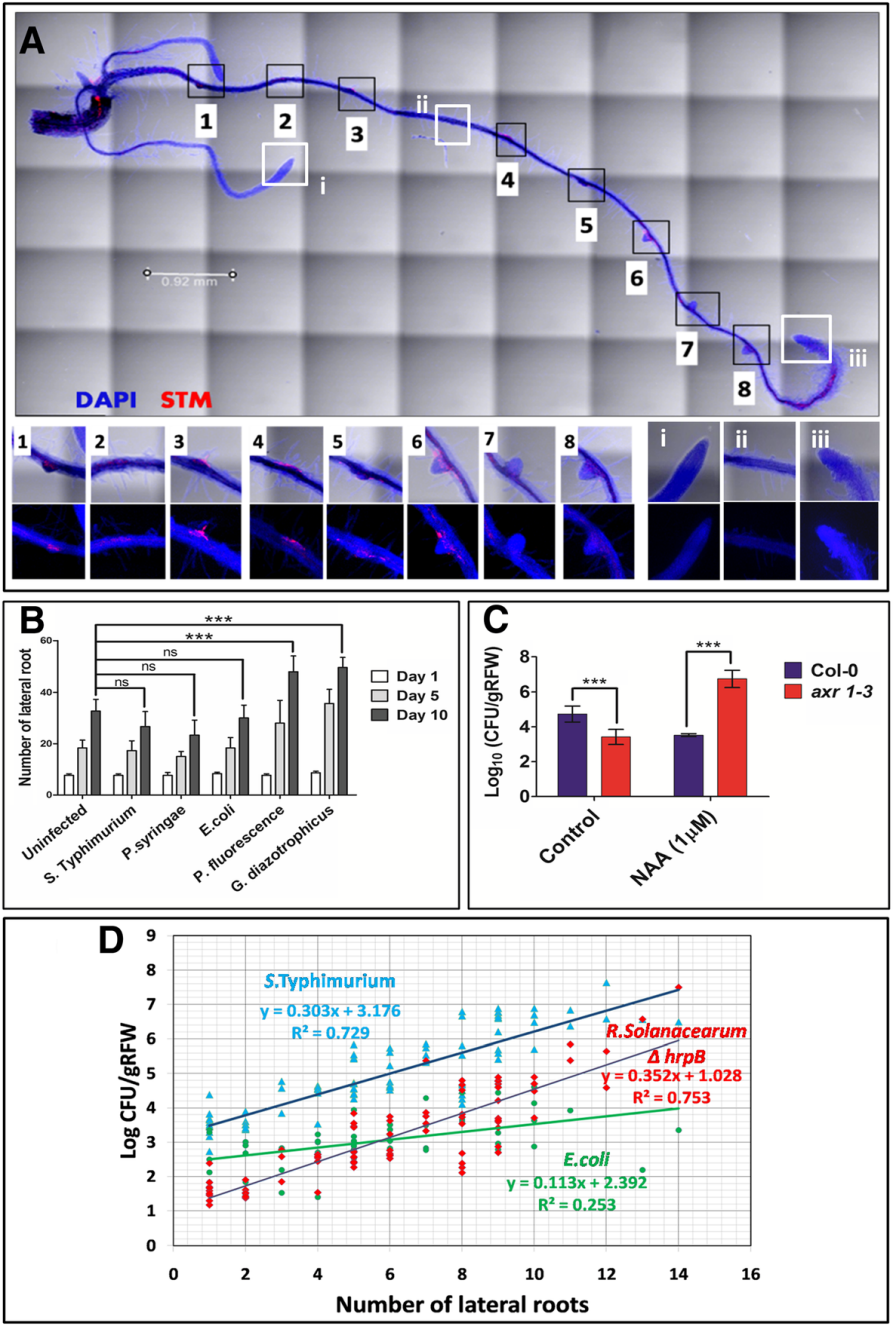
In-vitro colonization of *Salmonella* is dependent on lateral roots. (**a**) Representative image depicting *Salmonella* (mcherry tagged) colonization near the lateral root emerging area. Root is stained with DAPI. (**b**) Number of lateral roots upon inoculation of various organisms on Day 1, 5 and 10. ANOVA was used to analyze the data. The comparison shown is between uninfected and infected plants at 10th day. *** = *P* < 0.001; ns = not significant. (**c**) *Salmonella* colonization upon inhibition and induction of lateral roots by NAA for Col-0 and *axr 1–3* respectively. ANOVA was used to evaluate the significance. *** = *P* < 0.001. Each experiment was repeated 3 times with 5 replicate each. (**d**) Correlation of *Salmonella* colonization with lateral root (at 95% CI). *Ralstonia solanacearum ΔhrpB* was used as positive control and *E.coli* was used as non phytopathogenic bacteria control respectively. *N* = 50 for each organism for calculating correlation

Since plant cell wall and middle lamella are chemically composed of cellulose and pectin respectively, phytopathogens producing cellulases and pectinases can degrade and enter the host tissues [26]. In order to understand *Salmonella* mediated active degradation of these polysaccharides, we examine the growth profile of *Salmonella* in minimal media with cellulose and pectin as sole carbon source. Unlike plant pathogens, like *P. syringae, R. solanacearum* and *X. oryzae*, *Salmonella* is incapable of utilizing cellulose/pectin (Additional file 1: Figure S1A-B). Thus active invasion by degrading cell wall of the plant is not possible. Tomato roots inoculated with these organisms were observed to study pattern of colonization inside the root tissue. We observed that phytopathogens can cause tissue degradation but *Salmonella* cannot (Additional file 1: Figure S1C-E). Root cells in *Salmonella* and *Ralstonia* mutant Δ*hrpB* (deficint in type III secretion system) infected plants maintain their identity (Additional file 1: Figure S1E-F). Concomitantly, we observed higher CFU of phytopathogens like *Ralstonia*, *Xanthomonas* and *Pseudomonas* as compared to *Salmonella* in the root tissue but not in rhizoplane (Additional file 1: Figure S1G). We did not see any growth defect of these organisms in concentrated tomato root exudates (Additional file 1: Figure S1H), thus confirming that plant derived secretary metabolites is not responsible for lower CFU of *Salmonella*. Therefore, we examined the entire root thoroughly using tile scan in confocal microscope. We observed that there was very high colonization of *Salmonella* (Fig. 1a [1–8]) in the lateral root emerging regions as compared to the other regions (Fig. 1A [i-iii]). However, we did not find this pattern in phytopathogen *Ralstonia*, which equally colonizes the lateral root emerging and non-emerging sites (Additional file 1: Figure S1I). Beneficial organisms like mycorhizal fungi are known to enter through these lateral root emerging sites [27]. Plant growth promoting Rhizobacteria (PGPR) are known to enhance root biomass by producing various phyto-hormones and trigger root development [28]. However, to understand whether the *Salmonella* entry via LR is a chance event or triggered event, we counted the number of LR before and after *Salmonella* treatment and compared it with the un-inoculation control. Unlike PGPRs like *G. diazotrophicus* (endophyte) and *P. fluorescence* (rhizospheric and surface colonizing bacteria), *Salmonella* cannot induce LR formation in Arabidopsis (Fig. 1b).

To investigate the efficacy of lateral root dependent colonization, we used an Arabidopsis mutant *axr1–3* (from TAIR) which produces fewer lateral roots [29]. However, exogenous auxin can induce lateral roots in *axr1–3*. [29] We use naphthalene acetic acid (NAA), a synthetic auxin, to induce lateral root formation. Upon NAA treatment, in *axr1–3* we observed concentration dependent increase in number of lateral roots. Lateral roots in Col-0 on the other hand, increases but drops drastically beyond 100 nM NAA (Additional file 2: Figure S2A). These NAA treated roots were dipped in *Salmonella* suspension to analyze the colonization. Upon induction of lateral root in *axr1–3* with NAA, *Salmonella* colonization was significantly enhanced (Fig. 1c). On the contrary, Col-0 produced fewer lateral roots upon 1 μM NAA treatment, thus showing less colonization (Fig. 1c). We plotted endophytic CFU as a function of number of lateral root and observed a high correlation (R^2^ = 0.729 at 95% confidence interval (CI)) for *Salmonella*, but *E. coli* DH5α (non-phytopathogen control; R^2^ = 0.253 at 95%CI) had low correlation with the number of lateral roots (Fig. 1d). *P. syringae* also had lower colonization (phytopathogen control; R^2^ = 0.309 at 95%CI) (Additional file 2: Figure S2C). The low correlation for *P.syringae* could be due to its ability to degrade the cell wall and enter the root tissue through any part of the root (as an alternative to lateral root emerging sites). *Ralstonia* Δ*hrpB* endophytic CFU showed high correlation with number of lateral roots (Fig. 1d) and was used as a positive control (R^2^ = 0.762 at 95% CI). The rhizoplanic colonization (on the root surface) remains unaffected by the lateral root number for *Salmonella*, *E.coli* DH5α and *P.syringae* (Additional file 2: Figure S2D-F). Thus lateral root emergence can efficiently serve as gateway to the internal tissue of the plants for *Salmonella* without affecting its surface colonization.

### In-situ colonization of *Salmonella* is dependent on lateral roots

**Fig. 2:**
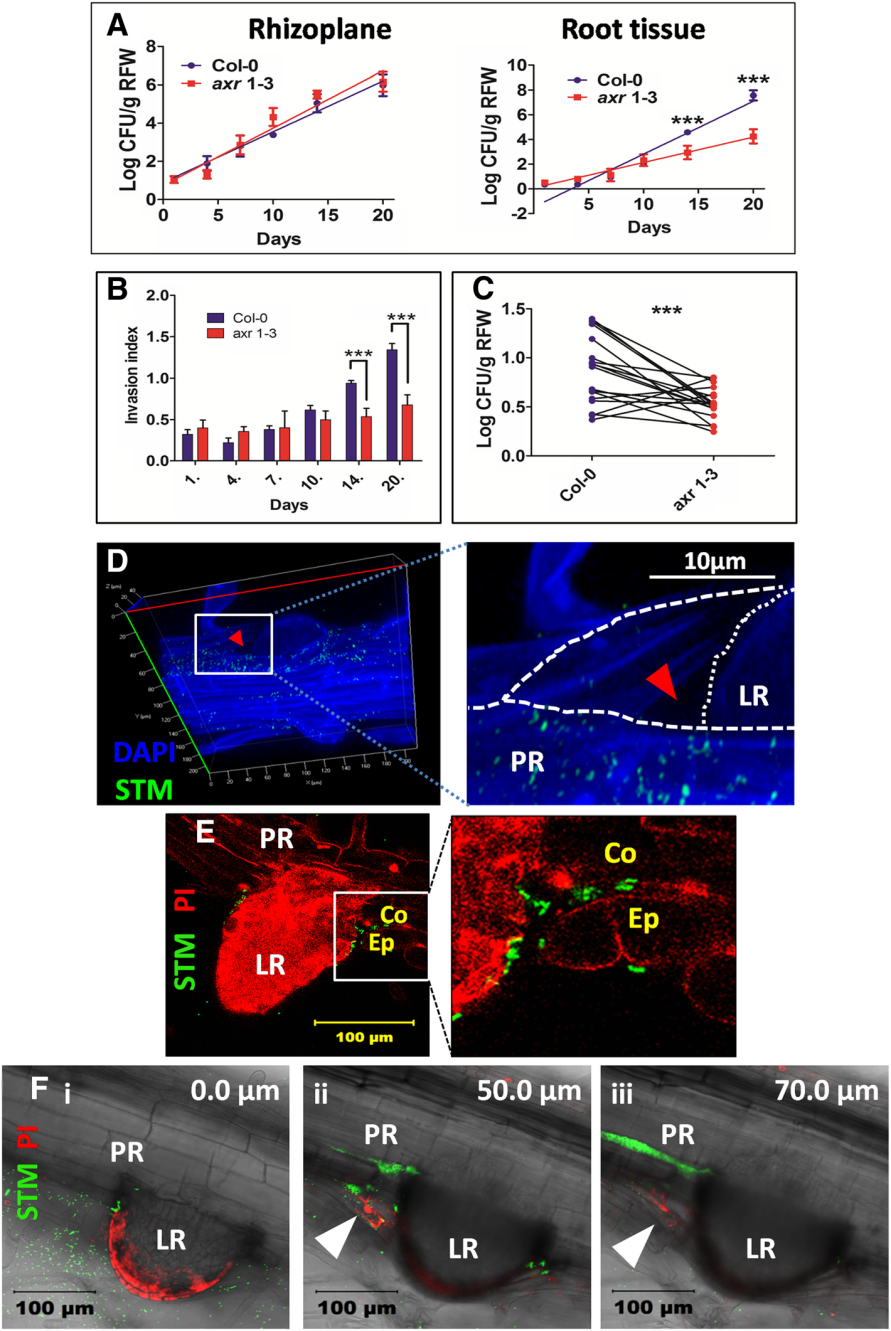
In-situ colonization of *Salmonella* is dependent on lateral roots. (**a**) Colonization of *Salmonella* in rhizoplane (left) and root tissue (right) of Col-0 and *axr1–3* grown in soil. ANOVA was used to analyze the data. *** = *P* < 0.001; ns = not significant. Best fit line was plotted (at 95% CI). (**b**) Invasion index (Log CFU root tissue/Log CFU rhizoplane) for Col-0 and *axr 1–3* was plotted over different days. One way ANOVA was used to evaluate the significance. *** = *P* < 0.001. Each experiment was repeated 3 times with 3 replicate each. (**c**) One-to-one correlation for root tissue colonization upon co-transplantation of Col-0 and *axr 1–3* in same pot containing *Salmonella* (10^8^cells/g soil). The experiment was repeated 3 times with 5 replicate each. Wilcoxan rank test was used to analyse the data. *** = P < 0.001. (**d**) Representative image showing remodeling of epidermis and lateral root emergence with *Salmonella* colonization. The red arrow head shows the space created when lateral root (LR) emerges from primary root (PR). (**e**) Representative image of transverse section of Arabidopsis root showing *Salmonella* in between the epidermis (Ep) and cortical cells (Co). (**f**) Transverse section of emerged lateral root in tomato with 3 representative Z-stacks: **i**. Surface view of lateral root (LR) and primary root (PR); **ii**. Remodeling of epidermis (White arrow head) and *Salmonella* entry through the space between PR and LR; **iii**. *Salmonella* colonization in between epidermis and cortical cell layer

Based on the in-vitro observations we carried out a similar experiment in soil with a modification that soil was pre-treated with *Salmonella* and 10 days old Col-0 or axr*1–3* seedlings were transplanted. The bacterial burden in rhizoplane and root tissue was assessed. Interestingly, the CFU in the rhizoplane for both Col-0 and *axr1–3* were not different, nevertheless, the CFU in root tissue was significantly higher for Col-0 after 14 and 20 days (Fig. 2a). A significant increase in invasion index (Log CFU root tissue/Log CFU rhizoplane) in a time dependent manner was observed for Col-0 but not for *axr1–3* (Fig. 2b). To further understand the role of lateral roots as an entry point, we co-transplanted Col-0 and axr*1–3* in same soil pretreated with *Salmonella*. Out of 20 experiments, data from 17 experiments were plotted and analyzed because either or both the pair was dead till 20th day in remaining pots. The mean CFU in root tissue for Col-0 was significantly higher as compared to *axr1–3* in same pot (Fig. 2c). Thus, irrespective of the plant (wild type or *axr1–3*) grown on same soil, *Salmonella* colonization depends solely on the number of lateral roots produced by the plant.

Lateral root originates from the pericycle cells because of auxin reflux between pericycle and endodermis [30] but its emergence requires cooperation from the neighboring cells like endodermis [31]. This leads to the weakening of paracellular junction of endodermis to allow lateral root to cross the endodermis. The auxin signal spreads from the lateral root to the cortex and epidermis resulting in coordinated remodeling of these layers [32]. Therefore, lateral root emergence is always accompanied by epidermis remodeling, wherein epidermis opens up for lateral root emergence [33].This results in, a cavity formation between primary root and lateral root (Fig. 2d; Additional file 3: Figure S3A-B).We took optical sections of the emerging lateral root, previously inoculated with GFP-tagged *Salmonella*, and a 3-D image was generated. The bacteria were located as deep as 52 μm which approximately correspond to the region between endodermis and pericycle in the root under observation (Additional file 3: Figure S3C-D). Transverse section of the arabidopsis root showed the entry of *Salmonella* through the remodeled epidermis and colonized the region between the epidermis and cortex (Fig. 2e). The same results were observed in soil grown tomato plants where *Salmonella* was able to colonize the lateral root emerging area (Fig. 2f i). Subsequent optical sections showed the epidermis remodeling and *Salmonella* entry between the epidermis and cortical cell layer (Fig. 2f ii and iii). Hence, we conclude that *Salmonella* can enter the root tissue via the opening created between main root and lateral root and can penetrate the deeper layer of the root.

### Mild salinity induced lateral root renders the plant susceptible to *Salmonella* invasion

**Fig. 3:**
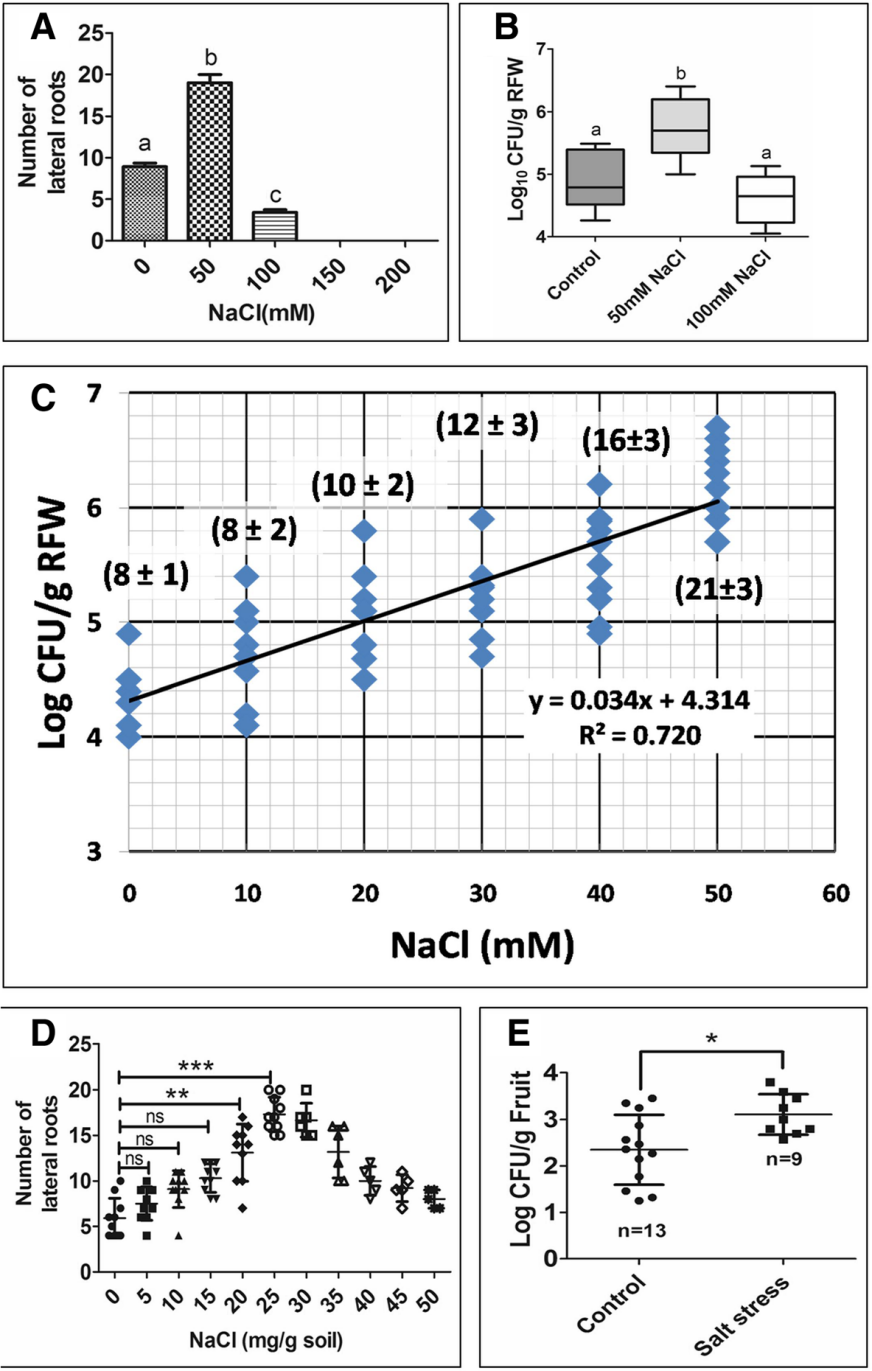
Salinity renders the plant susceptible to *Salmonella.* (**a**) Number of lateral roots upon treatment with varing amount of NaCl. (**b**) *Salmonella* colonization on arabidopsis roots treated with 50 mM and 100 mM NaCl. One way ANOVA followed by Bonferroni’s post test was used to analyse the data. Different letters shows significance at *P* < 0.05. (**c**) Correlation between salt stress and *Salmonella* colonization on arabidopsis root (at 95% CI). The values in parenthesis represent number of lateral root (Mean ± SD). (**d**) Number of lateral root of tomato plotted against varying salt stress. One way ANOVA followed by Bonferroni’s post test was used to analyse the data. *P* < 0.001 = ***; ns = not significant. (**e**) Tomato fruits infected with *Salmonella* upon mild salinity stress (25 mg NaCl/g soil; EC = 2.25dS/m). Each experiment was repeated 3 times with 3 replicate each. Mann-Whitney test was used to analyse the data. *P* < 0.05 = *

Since lateral roots facilitate the entry of *Salmonella* into the root tissues, we examined conditions which can induce lateral root formation. Abiotic stress factors like salinity are known to cause morphogenetic changes in root [34, 35]. In order to check the affect of salinity towards root development and colonization of *Salmonella*, 7 day old Arabidopsis seedlings were transferred to MS agar plate supplemented with varying concentrations of NaCl. Lateral root formation was significantly increased in Arabidopsis seedlings till 50 mM NaCl beyond which there was not much increase (Fig. 3a; Additional file 4: Figure S4). This was in accordance with increased CFU/g RFW (Fig. 3b). 100 mM NaCl treated plants were used as negative control as they we treated with stressor but do not induce lateral roots. Concomitantly, we observed an increase in CFU with varying salt concentrations in a dose dependent manner (Fig. 3c). In soil, we used varying concentration of NaCl to enhance the electrical conductivity and 20 days old tomato seedlings were transplanted. The number of lateral root increases with increase in NaCl concentration till 25 mg NaCl/g soil, beyond which it decreases (Fig. 3d). The concentration of NaCl used in this study (25 mg NaCl/g soil) corresponds to the electrical conductivity of 2.24mS/cm which is mild stress for tomato [36]. We observed increase in CFU as the salt concentration was raised from 0 to 25 mg/g soil (Additional file 5: Figure S5). We examined the fruits from infected plants for estimating *Salmonella* burden. The percentage of the fruits infected with *Salmonella* was higher in the plant grown in saline soil as compared to the control soil (Additional file 6: Figure S6). Fruits obtained from these plants had enhanced *Salmonella* burden than those grown on normal soil (Fig. 3e). Thus we conclude that salinity treatment increases the risk of *Salmonella* colonization on roots and its transmission to the fruits.

## Discussion

In the past few decades, the incidence of human infection by enteric bacteria through the consumption of contaminated salad has increased [37]. Plants can be contaminated during crop growth i.e.*,* before harvest through soil. In soil, *Salmonella* is known to survive for 300 days [38]. Greene et al reported that *Salmonella* enters tomato field through contaminated irrigation water [7]. Phytopathogen like *Ralstonia* and *Xanthomonas* produce cellulase (endoglucanse) [39, 40] and polygalacturonase (pectin methyl esterase and pectate lyase) secreted by their type III secretion system [41] and invade the plant root tissue. We screened phyto-pathogens like *Ralstonia solanacearum*, *Xanthomonas oryzae* and *Pseudomonas syringae* for their growth in cellulose or pectin as sole carbon source. These organisms degrade the plant tissues with cellulase or pectinase whereas *Salmonella* was unable to hydrolyze cellulose and pectin.

Pathogens utilize various natural openings to enter the host tissues. Leaves have specialized structures like stomata and hydathodes, but roots lack such natural openings. However epidermis remodeling during lateral root emergence can be utilized by microorganisms to gain access into the root tissues [24]. It was reported earlier that non-classical phytopathogens like *Salmonella enterica* and entero-pathgenic *E.coli* (O157:H7), colonizes and enter the root tissue via lateral root emerging region [22] and are known to induce defense responses in plants [42, 43]. Though observations were made that lateral root emergence can serve as entry point [22, 25, 44], the questions regarding the efficacy of the process and specific induction of lateral roots by the pathogen remained unanswered.

Auxin is indispensible for lateral root formation and indeed is a highly regulated developmental process in plants [45]. Lateral root initiation begins with auxin reflux between neighbouring cells and lateral root founder cell (LRFC) in pericycle which is dependent on PIN1 auxin transporter [30] but its emergence requires co-operation from the neighboring cells like endodermis [31]. This leads to weakening of paracellular junction of endodermis to allow lateral root to cross the endodermis. Local degradation of casparian strip and changes in its mechanical properties allows the lateral root to pass through endodermis [46]. The auxin signal spreads from the lateral root to the cortex and epidermis causing a coordinated remodeling of these layers [32]. We used Arabidopsis mutant *axr1–3* which produces fewer lateral roots, but could be induced to produce lateral root upon treatment with 1000 nM auxin [29]. We observed that irrespective of the number of lateral root, the rhizoplanic colonization remains unaffected. The endophytic population of *Salmonella* was high for Col-0 as compared to *axr1–3* (uninduced). When *axr1–3* was treated with 1000 nM NAA, the lateral roots increases significantly confirming that it is only the root tissue population which is dependent on the number lateral root (both in-vitro and in-situ), not the surface population. We screened phytopathogens like *Ralstonia solanacearum*, *Xanthomonas oryzae*, *Pectobacterium carotovorum* and *Pseudomonas syringae* and found that only *P.syringae* showed minimal killing of the host when inoculated on root. *P.syringae* also exibit high rhizoplanic colonization and tissue invasion (Additional file 7: Figure S7). *Ralstonia* inoculated plants, on the other hand, wilted and died because of the blockage of xylem. Comparatively high survival of *P.syringae* inoculated plants could be due to the fact that it is a foliar pathogen and root is not the primary site of infection. *E.coli* treated plants showed 100% survival and had low epiphytic as well as endophytic colonization (Additional file 7: Figure S7). Therefore, *P.syringae* was used as a phyto-pathogen control and *E.coli* as non-phytopathogen control. High correlation of endophytic *Salmonella* CFU and number of lateral roots (R^2^ = 0.729 at 95% CI) was observed. This implies *Salmonella* utilize lateral root emergence very efficiently to access the root tissues. In confocal microscopy phytopathogens were found to colonize both lateral root emerging and non-emerging site equally. Unlike *Ralstonia solanacearum,* the plant pathogen *P. syringae*, which colonize the root without killing the plant, was found to have a lower correlation (R^2^ = 0.309) with lateral root number. This suggests that phytopathogens which use lateral root as an alternative to the active invasion by degrading cell wall. *Salmonella* solely depends on the lateral roots to access the plant tissue, owing to its inability to degrade the cell wall. *Salmonella*, *E.coli* and *P.syringae* do not trigger the development of new lateral roots which can afftect the correlation. When Col-0 and *axr 1–3* were transplanted in the same pot containing *Salmonella* mixed with soil, we found enrichment of the pathogen in root tissue of Col-0 only. This supports the fact that given equal opportunity to colonize both Col-0 and *axr1–3*, *Salmonella* colonization in the root interior depends solely on the lateral root number. Further the presence of Col-0 in vicinity does not affect the colonization of the mutant *axr1–3*.

Beneficial microbes are known to modulate root growth and development [47]. We use *G. diazotrophicus,* an endophyte, [48] and *P.fluorescence*, a rhizospheric bacteria [49] as control organisms to monitor the affect on lateral root development. Treatment of plants with these organisms do not causes mortality (Additional file 7: Figure S7). Nevertheless, unlike these beneficial organisms, *Salmonella* do not promote lateral root development.

The growth of plants can be affected by various stress factors in soil [50]. Drought, salinity, sodicity, acidity and alkalinity are the major abiotic stresses in soil [51]. Salinity is caused by reduction in water potential due to the accumulation of dissolved ions and can lead to the accumulation of certain metabolites in plants, like glycine betains, proline which act as osmolytes [52]. Overcoming salinity is a challenging problem in agricultural fields. The reasons for salinity developement in soil are – excessive evapo-transpiration, injudicious use of chemical fertilizers, usage of irrigation water containing dissolved salts, etc. In Arabidopsis, mild salinity is known to cause morphogenetic changes in root system [35], however, high salinity is detrimental for the general development of the plant [53]. Decrease in primary root length and secondary root proliferation are well studied in this regard [35]. We chose to work with salinity stress because it promotes lateral root development (Additional file 4: Figure S4), which could be utilized by *Salmonella* to access entry into the root. Taken together our results suggest that plant roots not only become susceptible to *Salmonella* invasion but also enhance its transmission to the aerial edible organs. Various environmental and agronomic factors are known to influence the survival of *Salmonella* in soil like seasonal variation, cultivar, pathovar, residue from previous crop, irrigation regime [8, 54]. However, much was not know about the soil stress parameters. We, for the first time, tried to correlate the stress factors in soil with the invasiveness of the human pathogen in plants. Salinity induced lateral root proliferation and transmission of *Salmonella* to the edible organs are novel findings in this regard. Hence, outbreaks due to consumption of raw fruits and vegetables could be attributed to the soil stress factors in addition to climatic, agronomic and plant factors. However, the mechanism by which *Salmonella* overcomes the soil stress factors is not well understood. We would like to study the tri-partite interaction between *Salmonella*- plant and soil stress factors in future. Apart from the abiotic factors, biotic factors like Mycorrhiza and other PGPRs are also known to cause lateral root proliferation [27, 28]. We also aim to explore the possibilities of such beneficial organisms in affecting the colonization of *Salmonella* in roots.

## Conclusion

Pre-harvest contamination of edible plant products occur through soil. *Salmonella*, a human pathogen, is known to persist for about 300 days in soil [38]. The transmission of pathogen from soil to plant tissue is facilitated by the lateral root emerging areas. The remodeling of epidermis during lateral root emergence creates a cavity which can be utilized by *Salmonella* to invade the root tissue. This dependence on lateral root is not a general affect because phytopathogens do not solely depend on them, probably because of their ability to degrade cellulosic cell wall of plant. But *Salmonella* can utilize it as a gateway to access the deeper tissue of the roots, making it an opportunistic organism. It is known that environmental and soil factors can influence the colonization status of *Salmonella* [8]. Our data suggest that osmotic stress in soil by the dissolved salt can also affect the colonization of *Salmonella*. Mild salinity can cause morphogenetic changes in root architecture like enhancement of lateral roots, thus making more gateways for the pathogen to enter into the host tissue.

## Methods

### Growth conditions for plant and assessment of number of lateral roots

*Arabidopsis thaliana* ecotype Col-0 and mutant *axr 1–3* seeds were surface sterilized (0.01% *w*/*v* SDS with 70% *v*/v ethanol), placed on Murashige and Skoog (MS) agar plate and stratified for 4 days. The plates were incubated in a growth chamber (Geotech, Korea) with 16/8 h light and dark cycle at 22 °C and 75% relative humidity. After 7 days of germination, they were transferred to naphthelene acetic acid (NAA) containing plate (0, 50, 100, 500 and 1000 nM) and lateral roots were counted after 1 week. Another set of plants (14 days old) were transferred to NaCl containing plate (25, 50, 100, 200 mM) and assessed for lateral roots after 1 week. Seedlings were transferred to autoclaved potting mixture as and when required. The pots were kept in growth room at 22 °C. 16 h light /8 h dark cycle was used as photoperiod regime with a relative humidity of 75% and light intensity of ~ 6000 lx. Pots were irrigated with 15 ml sterile water every day.

### Bacterial strains, media, and culture conditions

Bacteria were plated either on LB medium or LB Supplemented with Ampicilin (50 μg/ml) (for *Salmonella* Typhimurium strain ATCC 14028 and *S.* Typhimurium expressing GFP/mcherry respectively [55]) from glycerol stocks. Plating onto Salmonella-Shigella agar (SS agar), a semi-selective differential medium, was used to determine *S.*Typhimurium as and when required. *Escheichia coli* DH5α (ATCC) [56], *Ralstonia solanacearum* strain F1C1 [57], *Pectobacterium carotovorum* ATCC 15713 and *Xanthomonas oryzae* pv oryzae strain 189 (kind gift from Prof. R Sonti, NIPGR, India) was grown on LB plates. *Ralstonia* strain expressing mcherrry (kind gift from Prof. SK Ray, Tezpur University, India) was grown on LB with 50 μg/ml gentamycin. *Gluconacetobacter diazotrophicus* SO01 (kind gift from Prof. N Earanna, UAS, Bangalore, India) was grown on LGIP media with 10% sucrose. *Pseudomonas syringae* pv.tomato DC3000 (ATCC-BAA 871) (kind gift from Prof. R Sonti, NIPGR, India) and *Pseudomonas fluorescence* ATCC 13525 was grown on King’s B medium [58] (kind gift from Prof. N Nagaraj, UAS, Bangalore, India). Bacterial cultures were grown at 30 °C (*P. syringae, P.fluorescence, R.solanacearum, X. oryzae, G. diazotrophicus, P. carotovorum*) for 36 h or at 37 °C (*S.* Typhimurium and *E.coli*) for 24 h in plates. Growth curve for *S.* Typhimurium, *P. syringae, R.solanacearum* and *X. oryzae* was done in M9 minimal media with 0.3% cellulose or pectin as sole carbon source.

### In-vitro Arabidopsis roots inoculation

Roots of 14-day old Col-0 or *axr 1–3* seedlings were dipped in 10^8^ cells/ml culture of *S.*Typhimurium, *P.syringae* or *E.coli* and incubated for 72 h under similar growth conditions. Roots were washed twice with 1X PBS to remove the un-adhered cells and homogenized using 1 mm glass beads in bead-beader (Biospec MiSci, USA). The suspension was serially diluted and plated on SS agar (for *Salmonella* and *E.coli*) or King’s B agar (for *P. syringae*). The CFU was normalized to root fresh weight (RFW).

### In-situ plant inoculation through soil

*Salmonella* Typhimurium was grown to an optical density (OD_600nm_) of 0.3, which corresponds to 10^8^ CFU/ml. Cells were centrifuged, re-suspended in 0.5X MS broth (only salt; without sucrose and hormones) and mixed thoroughly with the soil (1 ml suspension/g soil). The soil was allowed to dry and filled in pots. 10-day old Col-0 and *axr1–3* seedlings were transplanted in these soils. *Salmonella* burden was assessed in rhizoplane and root tissue after 1, 4, 7, 10, 14 and 20 days by the following procedure: After removal of the soil from the roots, they were put in sterile PBS and vortexed for 20 min to remove the surface-adhered rhizoplanic population. The rhizoplanic suspension was serially diluted and plated to assess the rhizoplanic population (Detailed procedure is mentioned in Additional file 8: Figure S8 and Additional file 9: Figure S9. The same root images before and after vortex are shown in Additional file 8: Figure S8B. The error percentage of the procedure was less than 0.001). The roots were immediately taken out from the suspension and crushed using mini bead-beader. The tissue homogenate was serially diluted and plated to assess the root tissue population. The CFU in rhizoplane and root tissue was normalized to root fresh weight (RFW). When surface sterilization of the root was done using 1% sodium hypochloride for 1 min, the bacteria inside the lateral root emerging region were killed because of the opening present at this area. Therefore, we use mechanical separation of the rhizoplanic population by vortexing. The viability of the bacteria localized inside the lateral root emerging region was confirmed by PI staining (Additional file 8: Figure S8C). This strategy was used only for arabidopsis roots. Due to high mechanical damage to the tomato roots, this method was not used and surface sterilization was performed with 1% bleach for 1 min. Arabidopsis roots, on the other hand were unharmed and on mechanical damage was observed (Additional file 8: Figure S8B). Invasion index (Log CFU root tissue/ Log CFU Rhizoplane) was calculated and plotted for each time point.

### Co-transplantation experiment

In another set both Col-0 and *axr1–3* were co-transplanted in the same pot pretreated with *Salmonella* and CFU in tissue was estimated after 20 days. Three such pots were examined and mean CFU of Col-0 and axr1–3 were plotted. Because of large variations, the experiment was repeated 20 times. One-to-one correlation was plotted with mean CFU in tissue for Col-0 and *axr1–3* obtained from each experiment. The paired values are the mean CFUs derived from plants from same pot. Wilcoxan matched pair test was used to analyze the data.

### Salinity treatment and *Salmonella* transmission to aerial organs

Tomato seeds were surface sterilized (with 1% bleach and washed with sterile water) and sown in autoclaved nursery soil. The soil in experimental pots was mixed with varying amount of NaCl (0, 25, 50, 75 and 100 mg NaCl/g soil). The electrical conductivity was measured using electrical conductivity meter and nursery grown tomato seedlings (20 days old) were transplanted in them. The pots were kept in green house at 26 °C, relative humidity of 70%. Pots were irrigated with 200 ml sterile water every day using micropipette, taking care to avoid splashing of soil and mixing of bacteria. Plants were uprooted carefully after 7 days without harming the root, washed and number of lateral root was counted. In another set soil mixed with 25 mg NaCl /g soil was transplanted with 20 days old tomato seedling and *Salmonella* was added to the soil via irrigation water after 7 days (10^8^ CFU/500 ml). Plants were allowed to grow either for 1 week or 3 months. From the 1 week old plants, root were harvested, surface sterilized with 1% bleach for 1 min, crushed using mortar pastel and the suspension was serially diluted and plated on SS agar to assess the *Salmonella* burden. From the 3 month old plant, fruit were harvested, surface sterilized with 5% bleach for 10 min and assessed for *Salmonella* burden by crushing using mortar-pastel and plating onto SS agar.

### Confocal imaging of roots

Two weeks old Arabidopsis roots (after 3 days post inoculation) were washed twice with PBS, dipped in propidium iodide or DAPI (0.01 μg/ml) for 2 min. They were again washed with PBS and mounted on a cover slip. For soil grown plants, 5 times washing was done by sterile PBS. Imaging was done by Zeiss confocal microscope (LSM meta710, Zeiss, Germany) and was analysed using ZEN 2009 Light edition software. ZEN 2012 black edition platform was used for creating 3D image. The GFP channel was then subjected to depth coding using the same platform to assess the bacterial signal from various depth of the root tissue (Additional file 3: Figure S3C-D). A boundary enclosing the remodeled epidermis was marked and three regions of interest were chosen (i, ii and iii). The location of the bacteria was analyzed using the rainbow color code with red representing the top layer and blue representing the deepest layer. Each color represents bacterial signal (GFP) from a particular depth. The signals from the enclosed region indicated bacteria inside the remodeled root architecture. Signals from outside the enclosed region are because of the bacteria present along the curvature of the root (Additional file 3: Figure S3D). Whole root imaging was done using Leica SP8 confocal microscope using tile scan. An average of 95 Z-stacks (1 μm interval) were taken for individual tiles (each square in Fig. 1a; Additional file 1: Figure S1B, with DIC image). 3D reconstitution and maximum intensity projection was constructed using LAS X software.

### Statistical analysis

To determine whether the average *Salmonella* populations in root differed between Col-0 and *axr 1–3*, populations were log transformed and unpaired t-tests with a Welch correction were performed using Graph Pad Prism. Two-way ANOVA was performed to check the significance for NAA treatment with number of lateral roots and CFU. Linear regression at 95% confidence interval (CI) was plotted using Graph pad Prism version 7. MATLAB 2016 was used to create scatter plot and generating correlation between CFU and lateral root. R^2^ < 0.666 was considered non significant as per Pearson’s correlation tables at *P* < 0.05 (*n* = 50 for each organism). One way ANOVA followed by Bonferroni’s post test was used for analyzing time dependent increase in invasion index and for in-vitro *Salmonella* colonization of arabidopsis under salinity stress. For co-transplantation experiment, the paired mean CFU values from same pot for Col-0 and *axr1–3* was plotted and one-to-one correlation was generated with Graph-pad Prism 7. Wilcoxan matched pair test was used to analyze the data. Mann-Whitney test was used for analyzing the CFUs obtained from tomato fruits.

## Additional files

Additional file 1: **Figure S1.** Growth of bacteria on cellulose and pectin containing media and pattern of colonization on plant roots. (A and B) Growth of *Salmonella, Pseudomonas, Ralstonia* and *Xanthomonas* on M9 minimal media with 0.3% cellulose or pectin as the sole carbon source respectively. Tomato roots inoculated with (C) *Ralstonia solanacearum* (mcherry), (D) *Pseudomonas syringae* (GFP), (E) *Salmonella* Typhimurium (GFP) and *R. solanacearum* Δ*hrpB* (mcherry). Images were taken after 3 days post infection. Propidium iodide and DAPI is used to stain the plant in (E) and (F) respectively. Pr = Pericycle and En = Endodermis. The dotted line represents the vasculature. (G) CFU of *Salmonella* and phyto-pathogens in rhizoplane and tissue. One way ANOVA was used to analyze the data. Different alphabets represent significance at *p* < 0.005. (H) Growth of *Salmonella* and phyto-pathogens in tomato root exudates. (I) Representative image of *Ralstonia solanacearum* colonization on arabidopsis. The green arrowhead represents lateral root emerging areas and the white arrowhead represents non emerging areas. Please note that mcherry fluorescence is coming from all over the root (i) specially in the vasculature (ii and iii). (TIF 1310 kb)

Additional file 2: **Figure S2.** Induction of lateral root by Naphthalene acetic acid (NAA) and correlation with CFU. (A) NAA concentration dependent decrease or increase in lateral root number in Col-0 and *axr 1–3* respectively. ANOVA was used to analyse the data. Different alphabets represent significance at p < 0.005. Scale bar =1 cm. (B) *Salmonella* CFU on roots treated with varying concentration of NAA. (C) Scatter plot showing *Pseudomonas syringae* CFU inside root tissue with respect to number of lateral roots. (D-F) Scatter plot showing *Salmonella* (D), *E.coli* DH5α (E) and *Pseudomonas syringae* (F) CFU on rhizoplane with respect to number of lateral root. (TIF 603 kb)

Additional file 3: **Figure S3.** Epidermis remodeling during lateral root emergence and bacterial colonization. (A) Representative image showing *Salmonella* entering the gap created between primary root and lateral root during remodeling of epidermis in transverse section. White arrow is representing the cavity. (B) Orthogonal sections showing the gap created in epidermis and bacterial entry in X-Y, Y-Z and X-Z planes. White arrow head shows the site of entry. (C) Representative image showing remodeled epidermis (marked by dotted line) and *Salmonella* (GFP tagged) cluster inside the region (red arrow head). (D) Depth coding was done only for GFP channel to estimate the location of *Salmonella* inside the remodeled epidermis. The rainbow color coded chart was used to locate the bacteria at various depth. (i, ii and iii are three region of interest showing bacteria at different depth. PR represents primary root and LR represents lateral root. (TIF 1058 kb)

Additional file 4: **Figure S4.** Representative Images for lateral root phenotype for normal and 50 mM NaCl stress. Values in parenthesis represent number of lateral roots ±SD. Image was taken by Olympus STYLUS VH520 camera. Newly emerged lateral roots that were very small were observed by Olympus SZX7 stereoscope. (TIF 497 kb)

Additional file 5: **Figure S5.** Correlation between varying salt stress in soil with *Salmonella* colonization in root (at 95% CI). (TIF 154 kb)

Additional file 6: **Figure S6.** Percent fruit infected with *Salmonella* upon salt stress (25 mg NaCl/g Soil).and control condition. Student’s t-test was used to analyze the data. *** = *P* < 0.001; ** = *P* < 0.01. (TIF 88 kb)

Additional file 7: **Figure S7.** Parameters studied to chose phyto-pathogen and non-phyto-pathogen control. (A) Representative image of plant grown in soil inoculated with different bacterial strans. Images were taken by Olympus STYLUS VH520 camera after 7 days of transplantation. (B) CFU of bacteria in the root tissue after 3 days of infection. (C) Survival curve of plants grown on soil mixed with various organisms (*n* = 60). (D) Epiphytic colonization of tomato roots with *Salmonella*, *E.coli* and *P. syringae*. (E) Parameter used for selecting the good colonist and poor colonist for comparison with *Salmonella*. *E.coli* was selected as poor colonist whereas *P.syringae* was used as good colonist. (TIF 1109 kb)

Additional file 8: **Figure S8.** Protocol for separation of rhizoplanic bacteria from internalized bacteria. (A) Scematic representation of the protocol for estimating surface colonizing versus the invading bacteria. (B) Representative confocal image of the entire root before and after vortex. Note that these are same root shown before and after vortex. The same region on both the roots are zoomed in (A and B). Vortex above 200RPM leads to mechanical damage to the root. (C and D) Comparison between the viability of bacteria after vortexing and after 1% sodium hypochloride treatment respectively. Please note that the same root was first imaged after vortexing and then after sodium hypochloride treatment. Arrow head showing the internalized *Salmonella*. Ep = Epidermis; Co = Cortex. (TIF 2016 kb)

Additional file 9: **Figure S9.** Standardization and verification of time for vortexing procedure for separation of rhizoplanic bacteria. (A) Schematic showing the verification of the process of separation of rhizoplanic population. (B) Minimum time required for vortexing (‘X’) the root to isolate the surface adhering population from the rhizoplane. The CFU and the dilution factor are shown for stage 2 (C) Cross examination of the vortexed root (after 20 min). The roots were placed in fresh PBS again and vortexed for 5 more min and the suspension was plated. The CFU values at 0th dilution and colonies on plate are shown in table. Plates with colonies between 30 to 300 were counted. (D) Error in the process was calculated by [CFU at stage 2 (at 0th dilution)/(CFU at stage 1+ stage 2)} expressed in percentage. Note that X = 20 min was taken for all organisms. (TIF 459 kb)
