# Supplementary figures and images for: Root mediated uptake of Salmonella is different from phyto-pathogen and associated with the colonization of edible organs
Source: BMC Plant Biol. 2018 Dec 11;18:344. doi: 10.1186/s12870-018-1578-9 (PMC6290541; doi:10.1186/s12870-018-1578-9)

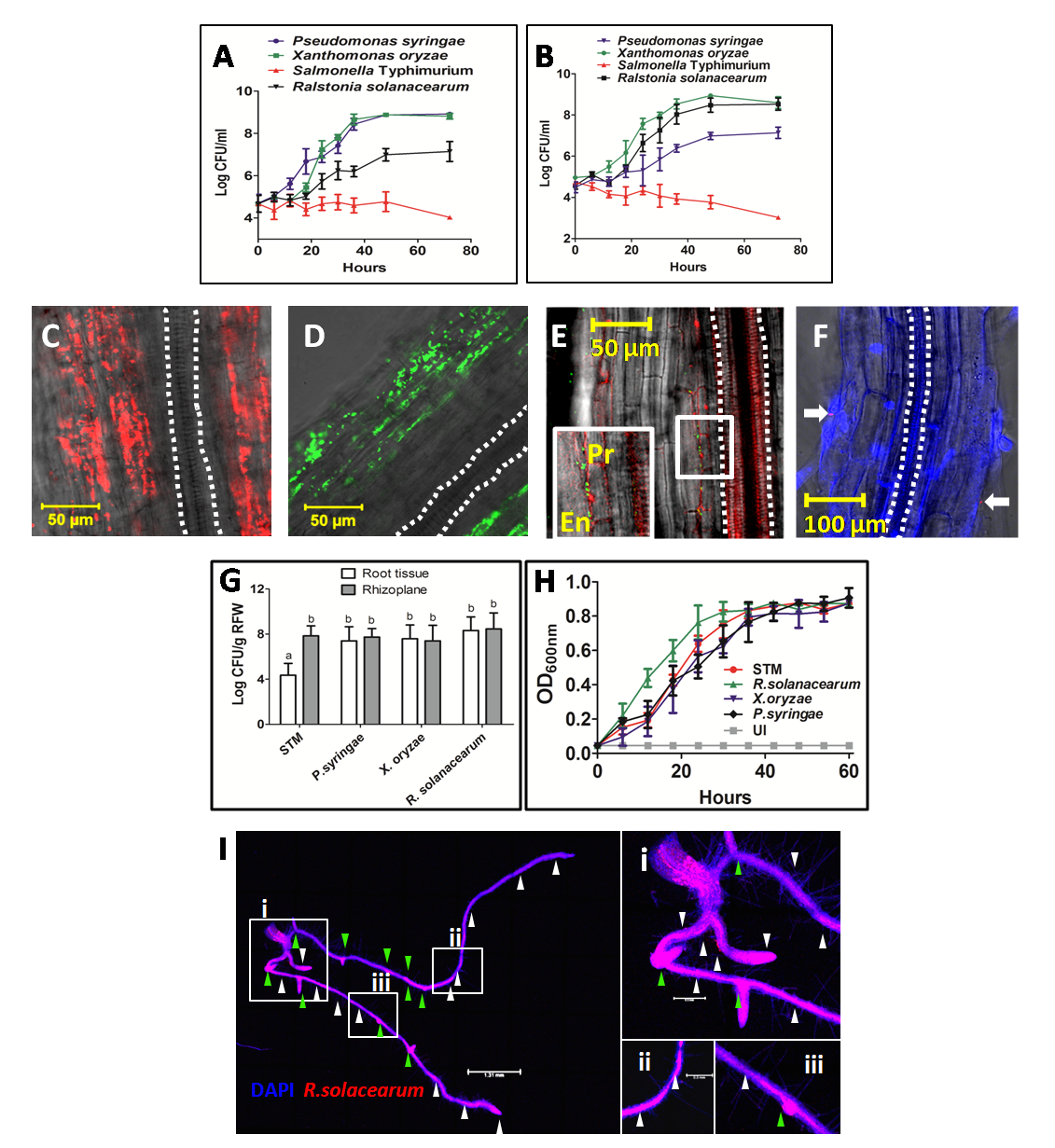

Supplement: Supplementary file 1 — Figure S1. Growth of bacteria on cellulose and pectin containing media and pattern of colonization on plant roots. (A and B) Growth of Salmonella, Pseudomonas, Ralstonia and Xanthomonas on M9 minimal media with 0.3% cellulose or pectin as the sole carbon source respectively. Tomato roots inoculated with (C) Ralstonia solanacearum (mcherry), (D) Pseudomonas syringae (GFP), (E) Salmonella Typhimurium (GFP) and R. solanacearum ΔhrpB (mcherry). Images were taken after 3 days post infection. Propidium iodide and DAPI is used to stain the plant in (E) and (F) respectively. Pr = Pericycle and En = Endodermis. The dotted line represents the vasculature. (G) CFU of Salmonella and phyto-pathogens in rhizoplane and tissue. One way ANOVA was used to analyze the data. Different alphabets represent significance at p < 0.005. (H) Growth of Salmonella and phyto-pathogens in tomato root exudates. (I) Representative image of Ralstonia solanacearum colonization on arabidopsis. The green arrowhead represents lateral root emerging areas and the white arrowhead represents non emerging areas. Please note that mcherry fluorescence is coming from all over the root (i) specially in the vasculature (ii and iii). (TIF 1310 kb) [file 12870_2018_1578_MOESM1_ESM.tif]

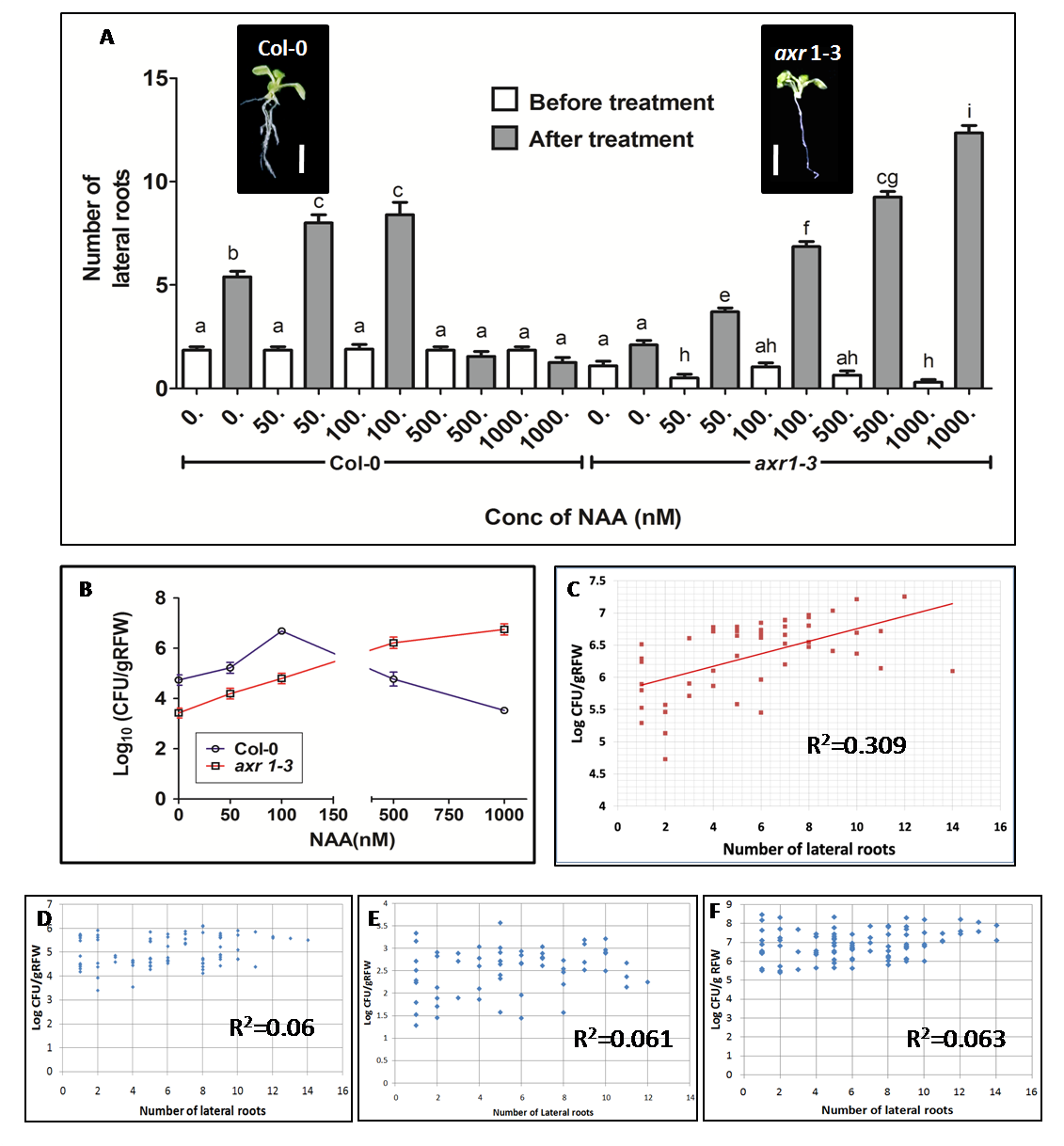

Supplement: Supplementary file 2 — Figure S2. Induction of lateral root by Naphthalene acetic acid (NAA) and correlation with CFU. (A) NAA concentration dependent decrease or increase in lateral root number in Col-0 and axr 1–3 respectively. ANOVA was used to analyse the data. Different alphabets represent significance at p < 0.005. Scale bar =1 cm. (B) Salmonella CFU on roots treated with varying concentration of NAA. (C) Scatter plot showing Pseudomonas syringae CFU inside root tissue with respect to number of lateral roots. (D-F) Scatter plot showing Salmonella (D), E.coli DH5α (E) and Pseudomonas syringae (F) CFU on rhizoplane with respect to number of lateral root. (TIF 603 kb) [file 12870_2018_1578_MOESM2_ESM.tif]

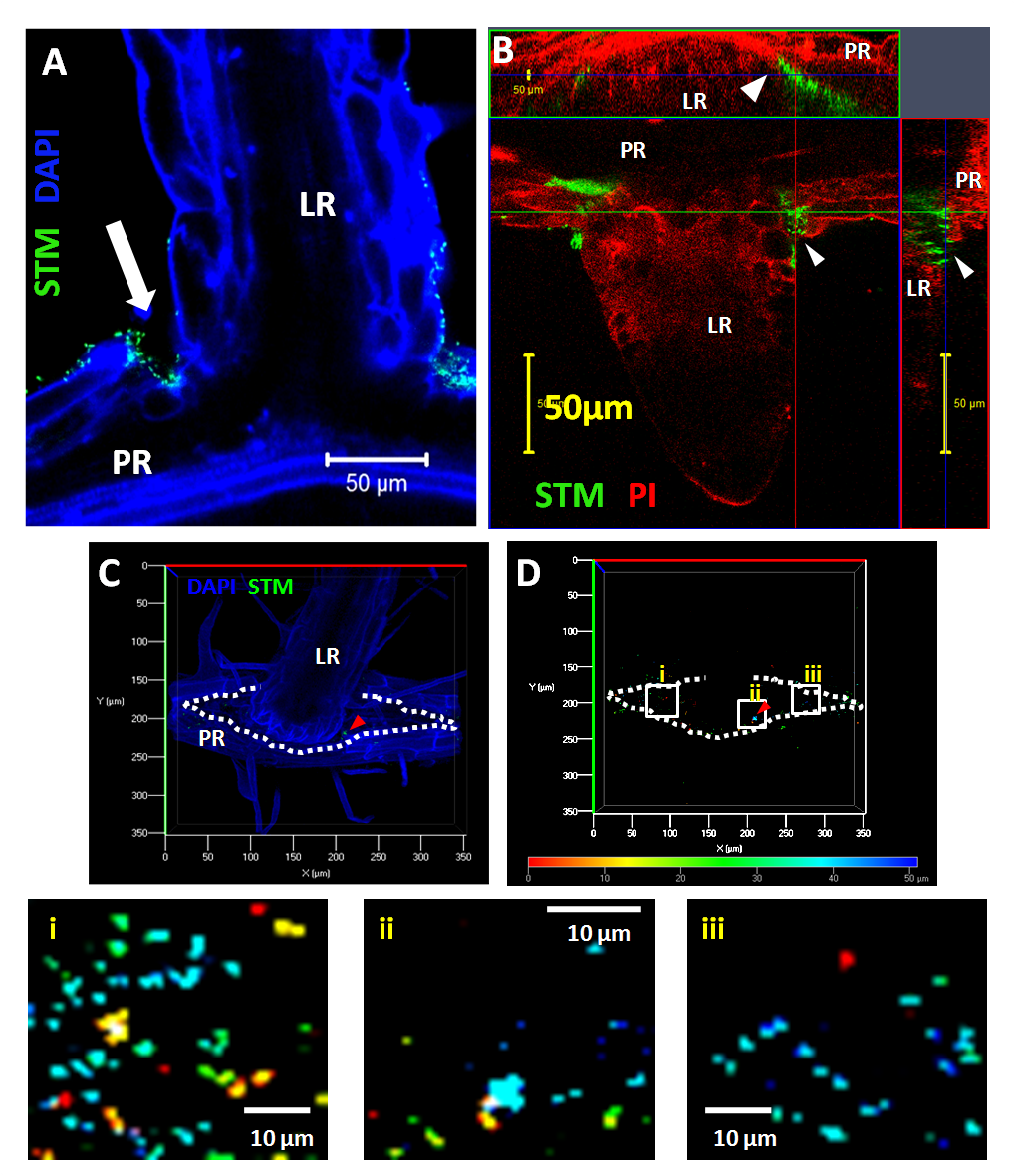

Supplement: Supplementary file 3 — Figure S3. Epidermis remodeling during lateral root emergence and bacterial colonization. (A) Representative image showing Salmonella entering the gap created between primary root and lateral root during remodeling of epidermis in transverse section. White arrow is representing the cavity. (B) Orthogonal sections showing the gap created in epidermis and bacterial entry in X-Y, Y-Z and X-Z planes. White arrow head shows the site of entry. (C) Representative image showing remodeled epidermis (marked by dotted line) and Salmonella (GFP tagged) cluster inside the region (red arrow head). (D) Depth coding was done only for GFP channel to estimate the location of Salmonella inside the remodeled epidermis. The rainbow color coded chart was used to locate the bacteria at various depth. (i, ii and iii are three region of interest showing bacteria at different depth. PR represents primary root and LR represents lateral root. (TIF 1058 kb) [file 12870_2018_1578_MOESM3_ESM.tif]

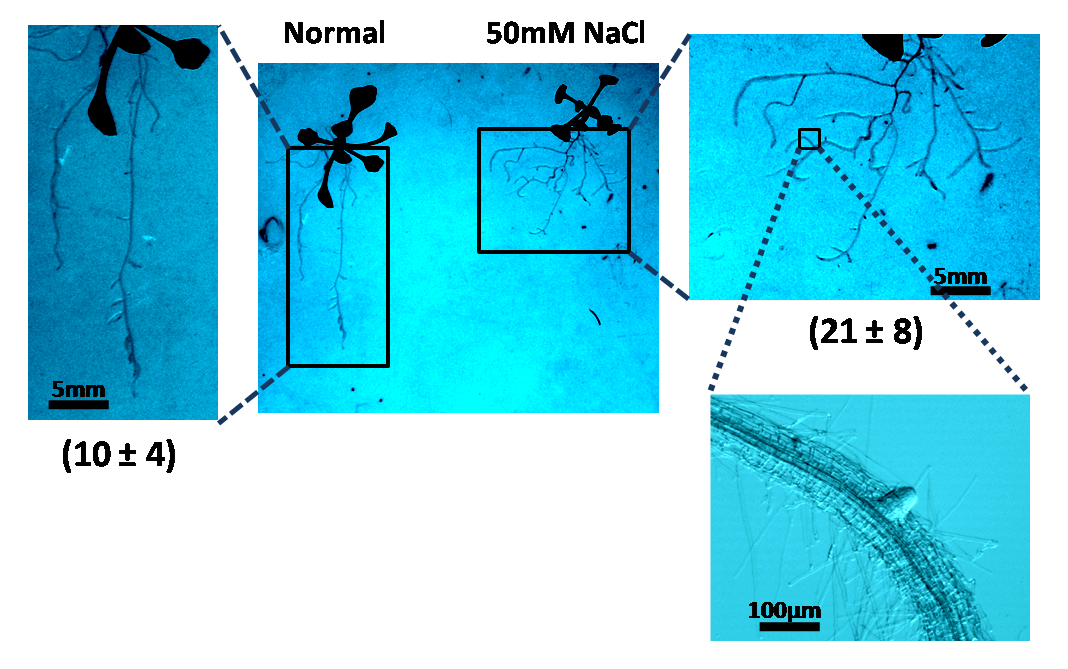

Supplement: Supplementary file 4 — Figure S4. Representative Images for lateral root phenotype for normal and 50 mM NaCl stress. Values in parenthesis represent number of lateral roots ±SD. Image was taken by Olympus STYLUS VH520 camera. Newly emerged lateral roots that were very small were observed by Olympus SZX7 stereoscope. (TIF 497 kb) [file 12870_2018_1578_MOESM4_ESM.tif]

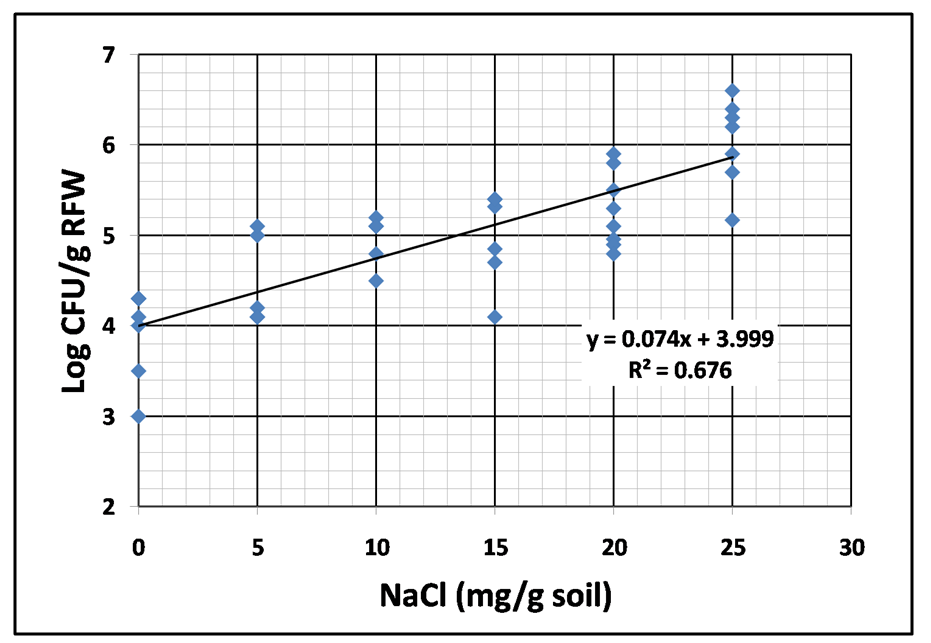

Supplement: Supplementary file 5 — Figure S5. Correlation between varying salt stress in soil with Salmonella colonization in root (at 95% CI). (TIF 154 kb) [file 12870_2018_1578_MOESM5_ESM.tif]

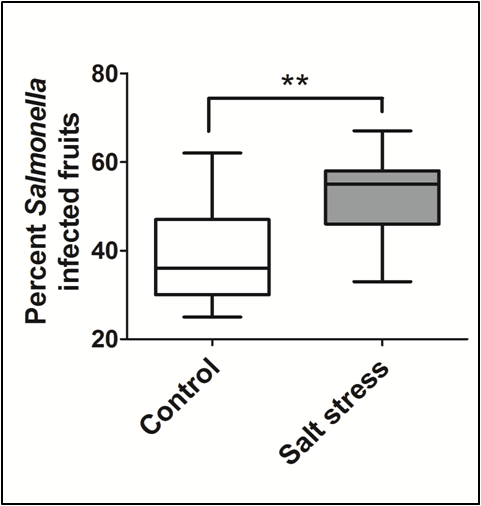

Supplement: Supplementary file 6 — Figure S6. Percent fruit infected with Salmonella upon salt stress (25 mg NaCl/g Soil).and control condition. Student’s t-test was used to analyze the data. *** = P < 0.001; ** = P < 0.01. (TIF 88 kb) [file 12870_2018_1578_MOESM6_ESM.tif]

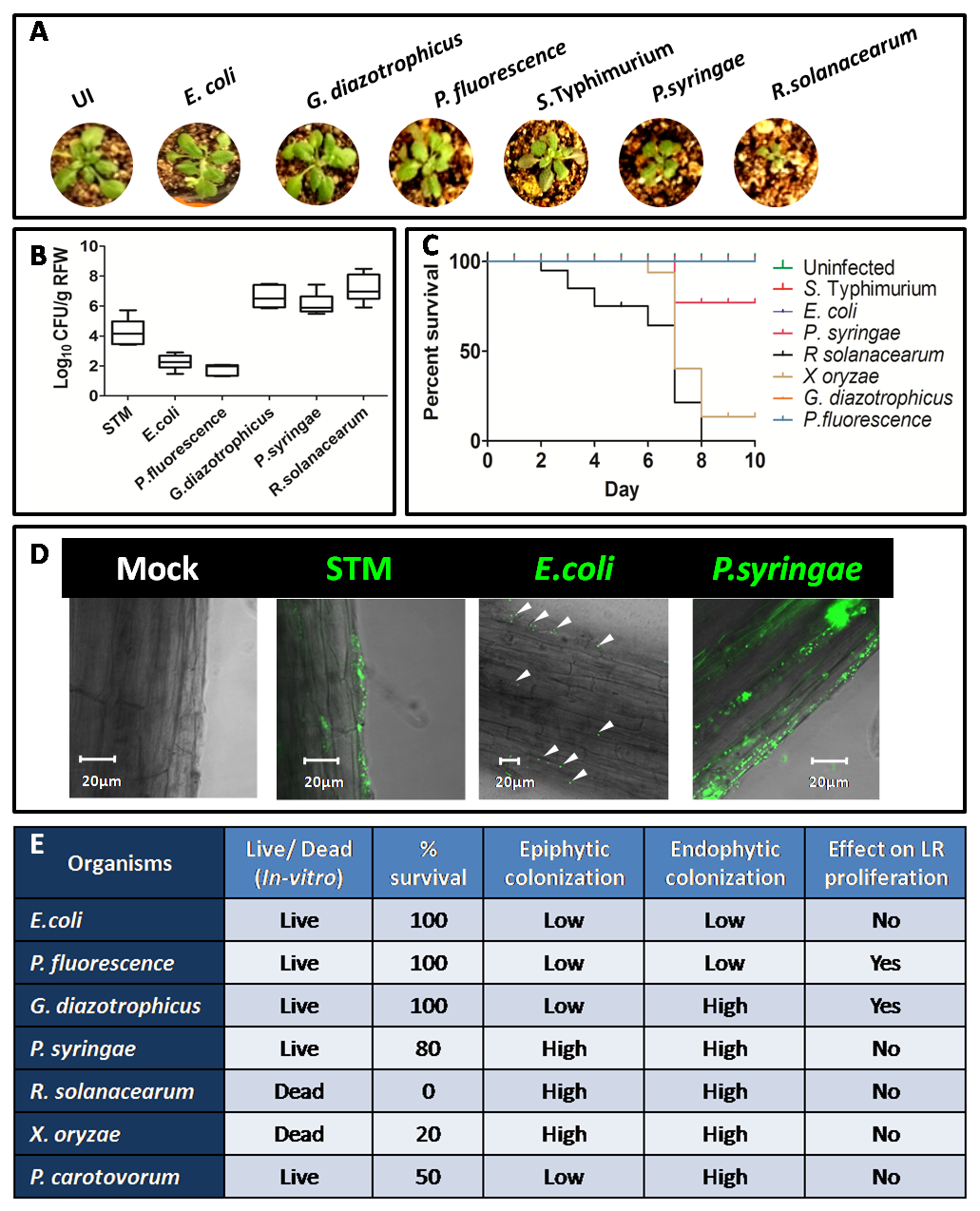

Supplement: Supplementary file 7 — Figure S7. Parameters studied to chose phyto-pathogen and non-phyto-pathogen control. (A) Representative image of plant grown in soil inoculated with different bacterial strans. Images were taken by Olympus STYLUS VH520 camera after 7 days of transplantation. (B) CFU of bacteria in the root tissue after 3 days of infection. (C) Survival curve of plants grown on soil mixed with various organisms (n = 60). (D) Epiphytic colonization of tomato roots with Salmonella, E.coli and P. syringae. (E) Parameter used for selecting the good colonist and poor colonist for comparison with Salmonella. E.coli was selected as poor colonist whereas P.syringae was used as good colonist. (TIF 1109 kb) [file 12870_2018_1578_MOESM7_ESM.tif]

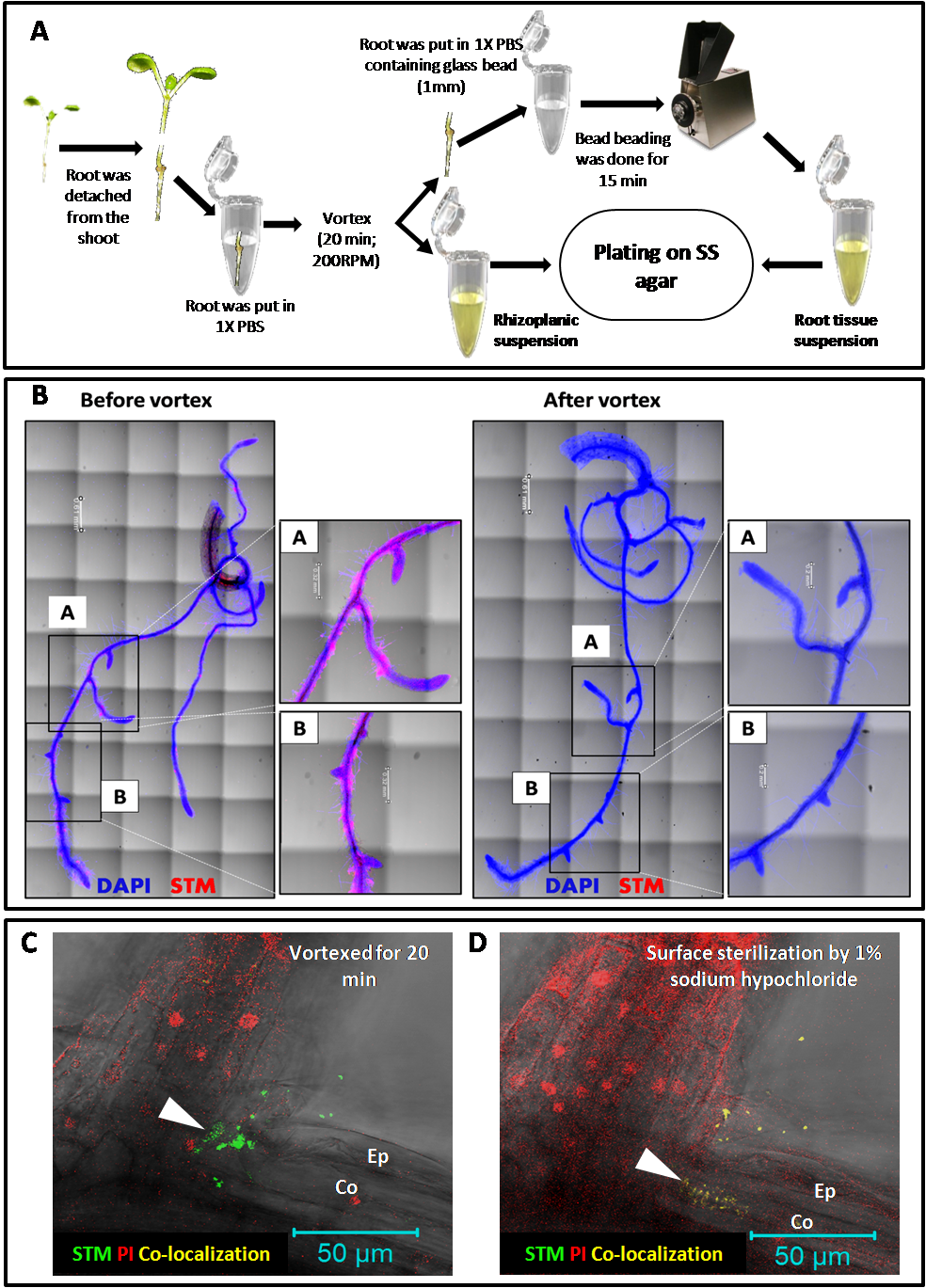

Supplement: Supplementary file 8 — Figure S8. Protocol for separation of rhizoplanic bacteria from internalized bacteria. (A) Scematic representation of the protocol for estimating surface colonizing versus the invading bacteria. (B) Representative confocal image of the entire root before and after vortex. Note that these are same root shown before and after vortex. The same region on both the roots are zoomed in (A and B). Vortex above 200RPM leads to mechanical damage to the root. (C and D) Comparison between the viability of bacteria after vortexing and after 1% sodium hypochloride treatment respectively. Please note that the same root was first imaged after vortexing and then after sodium hypochloride treatment. Arrow head showing the internalized Salmonella. Ep = Epidermis; Co = Cortex. (TIF 2016 kb) [file 12870_2018_1578_MOESM8_ESM.tif]

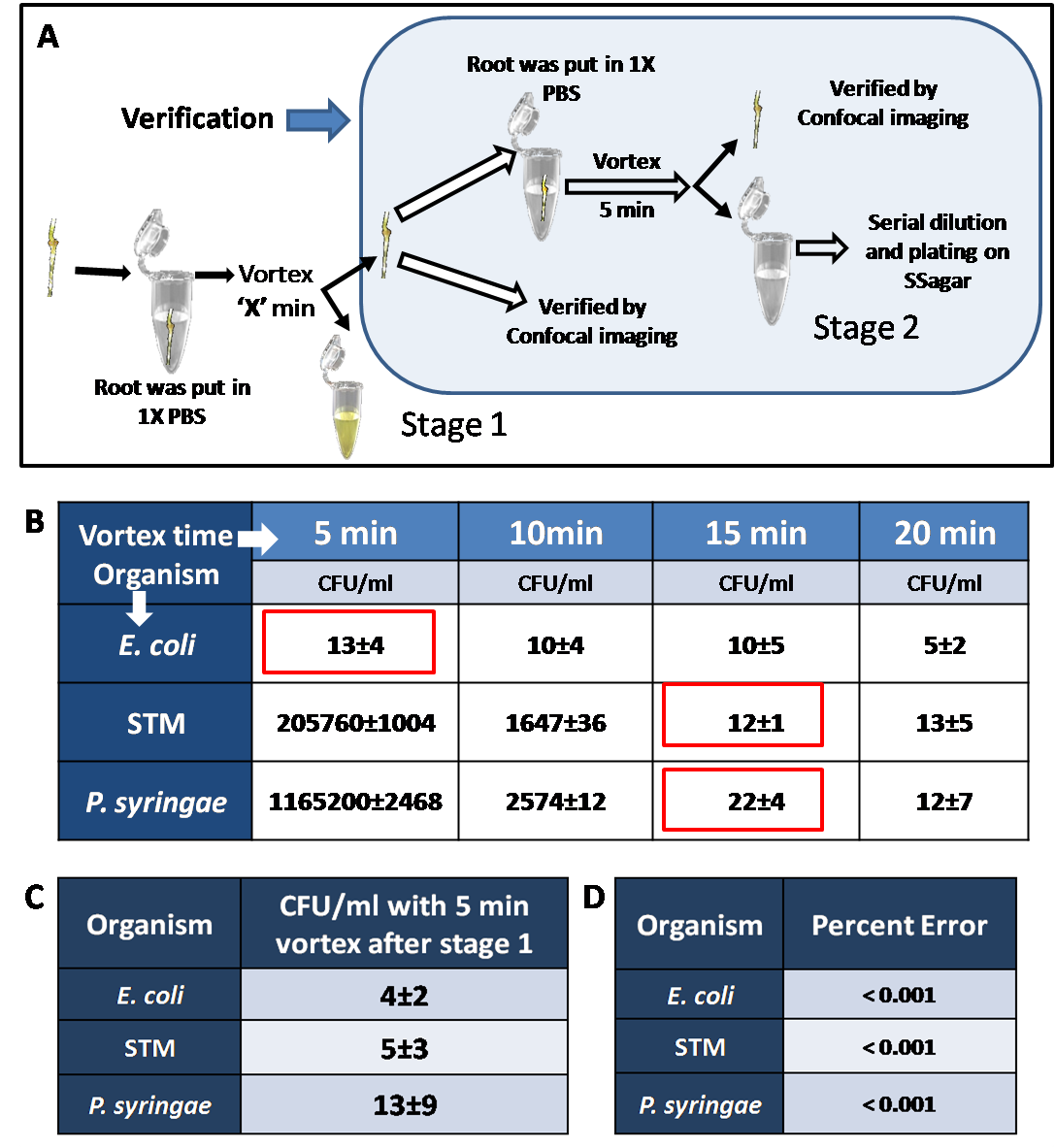

Supplement: Supplementary file 9 — Figure S9. Standardization and verification of time for vortexing procedure for separation of rhizoplanic bacteria. (A) Schematic showing the verification of the process of separation of rhizoplanic population. (B) Minimum time required for vortexing (‘X’) the root to isolate the surface adhering population from the rhizoplane. The CFU and the dilution factor are shown for stage 2 (C) Cross examination of the vortexed root (after 20 min). The roots were placed in fresh PBS again and vortexed for 5 more min and the suspension was plated. The CFU values at 0th dilution and colonies on plate are shown in table. Plates with colonies between 30 to 300 were counted. (D) Error in the process was calculated by [CFU at stage 2 (at 0th dilution)/(CFU at stage 1+ stage 2)} expressed in percentage. Note that X = 20 min was taken for all organisms. (TIF 459 kb) [file 12870_2018_1578_MOESM9_ESM.tif]
